# Molecular genetic and morphological characteristics of Micractinium thermotolerans and M. inermum (Trebouxiophyceae, Chlorophyta) from pyroclastic deposits of the Kamchatka Peninsula (Russia)

**DOI:** 10.18699/vjgb-24-79

**Published:** 2024-11

**Authors:** R.Z. Sushchenko, V.Yu. Nikulin, V.B. Bagmet, A.Yu. Nikulin

**Affiliations:** Federal Scientific Center of the East Asia Terrestrial Biodiversity of the Far Eastern Branch of the Russian Academy of Sciences, Vladivostok, Russia; Federal Scientific Center of the East Asia Terrestrial Biodiversity of the Far Eastern Branch of the Russian Academy of Sciences, Vladivostok, Russia; Federal Scientific Center of the East Asia Terrestrial Biodiversity of the Far Eastern Branch of the Russian Academy of Sciences, Vladivostok, Russia; Federal Scientific Center of the East Asia Terrestrial Biodiversity of the Far Eastern Branch of the Russian Academy of Sciences, Vladivostok, Russia

**Keywords:** microalgae, floristic findings, integrative approach, morphology, phylogeny, secondary structure of ITS rRNA, микроводоросли, флористические находки, комплексный подход, морфология, филогения, вторичная структура ITS рРНК

## Abstract

During the study of algal diversity in pyroclastic deposits of the Kamchatka Peninsula, Chlorella-like green algae strains VCA-72 and VCA-93 were isolated from samples collected from along the Baydarnaya river bed on the Shiveluch volcano in 2018 and at the outlet of thermal vapors along the edge of the caldera on the southern slope of the Gorely volcano in 2020. Identification of the strains was carried out within the framework of an integrative approach using microscopic and molecular genetic methods, including preliminary taxon identification, obtaining nucleotide sequences of the small subunit and the internal transcribed spacer rRNA, reconstruction of phylogenetic trees and secondary structures of the ITS1 and ITS2 rRNA regions. On the phylogenetic tree, strain VCA-93 was clustered in the Micractinium thermotolerans species clade. No differences were found when comparing the helical domain models of ITS1 and ITS2 in M. thermotolerans. Strain VCA-72 occupied a basal position in the M. inermum clade. The secondary structure patterns of the helices of strain VCA-72 were generally similar to those of M. inermum, but intraspecific variability was noted, mainly due to substitutions in the apical and lateral loops. Five hCBC substitutions were found in the helical regions of the studied M. inermum strains, while no CBC substitutions were found. A detailed analysis of morphology and life cycle allowed us to identify the characteristics of the cells in aging cultures: their size was significantly higher than in vegetative ones and they were pear-shaped, oval, and ellipsoidal with a shallow, wide constriction in the center. In addition, cells with colorless lipid droplets were detected in aging cultures of both species. The ability to synthesize and accumulate lipids indicates the great potential of the strains for the production of biodiesel fuel. A review of the habitats of previous and new findings allowed us to note the ecological plasticity of the studied species. The results obtained complement the information on the biogeography of the species: this is the first record of M. inermum for the territory of Russia, and that of M. thermotolerans, for the Kamchatka Peninsula.

## Abstract (ru)

В ходе исследования разнообразия водорослей пирокластических отложений вулканов Шивелуч и Горелый (полуостров Камчатка) были выделены Chlorella-подобные штаммы зеленых водорослей VCA-72 и VCA- 93 из проб, отобранных вдоль русла р. Байдарная на вулкане Шивелуч и на выходе термальных паров по краю кальдеры на южном склоне вулкана Горелый в 2018 и 2020 гг. соответственно. Идентификация штаммов выполнялась в рамках комплексного подхода микроскопическими и молекулярно-генетическими методами, включаю-щими предварительную идентификацию, получение нуклеотидных последовательностей малой субъединицы и внутреннего транскрибируемого спейсера рРНК, построение филогенетических деревьев и вторичных структур участков ITS1 и ITS2 рРНК. На филогенетическом древе штамм VCA-93 кластеризовался в видовой кладе Micractinium thermotolerans. При сравнении моделей спиральных доменов ITS1 и ITS2 у M. thermotolerans различий не выявлено. Штамм VCA-72 занимал базальное положение в кладе M. inermum. Модели вторичной структуры спиралей штамма VCA-72 в целом были сходны с моделями для M. inermum, однако отмечена внутривидовая вариабельность, обусловленная в основном заменами в верхушечных и боковых петлях. В спиральных участках исследуемых штаммов M. inermum обнаружено пять замен hCBC, тогда как замен CBC обнаружено не было. Детальный анализ морфологии и жизненного цикла позволил выявить в стареющих культурах клетки, которые по размеру значительно превышают вегетативные и имеют грушевидную, овальную и эллипсоидную формы с неглубоким широким сужением в центре. Также в стареющих культурах обоих видов были выявлены клетки с бесцветными липидными каплями. Способность синтезировать и накапливать липиды говорит о большом потенциале штаммов для производства биодизельного топлива. Обзор местообитаний предыдущих и новых находок позволяет сделать вывод об экологической пластичности исследуемых видов. Полученные результаты дополняют сведения о биогеографии видов: M. inermum обнаружен впервые на территории России, а M. thermotolerans – на полуострове Камчатка.

## Introduction

Micractinium Fresenius (Trebouxiophyceae, Chlorophyta) is a genus comprising Chlorella-like green algae, including symbiotic (M. conductrix (Brandt) Pröschold et Darienko, M. tetrahymenae Pröschold, Pitsch et Darienko) and freeliving organisms. The genus currently comprises 24 species (Guiry M.D., Guiry G.M., 2024). M. pusillum Fresenius is the type species. It is characterized by a coccoid organization and the formation of colonies of 2–4 cells and bristles (Fresenius, 1858). It was assumed that the ability to form colonies and the presence of bristles are distinctive features of this species (Komárek, Fott, 1983). However, it was shown that these traits only appeared as a protective mechanism in response to cocultivation with the rotifer Brachionus calyciflorus Pallas; in the absence of algophages, the features did not appear (Luo et al., 2005, 2006). In addition, the presence of zooplankton did not always result in the formation of bristles and colonies in Micractinium species (Pröschold et al., 2011). For example, M. conductrix, M. inermum Hoshina et Fujiwara and other species of the genus do not have bristles and are morphologically similar to algae of the genus Chlorella (Pröschold et al., 2011; Hoshina, Fujiwara, 2013; Hong et al., 2015).

Homoplastic characters leading to similarities between the genera Chlorella and Micractinium make it difficult to identify taxa using only morphological data. The use of an integrative approach, combining traditional microscopy methods and molecular phylogenetic analysis, allows to distinguish not only taxa poor in diagnostic characters, but also cryptic species (Komárek et al., 2014; Darienko, Pröschold, 2019).

Members of the genus Micractinium are well-known objects of biotechnology research. F. Quintas-Nunes et al. (2023) showed the growth-stimulating effect of exudates of Micractinium sp. NFX-FRZ on tomato plants. This could be due to phytohormones synthesized by the algae. M. inermum F014 was found to be able to treat radioactive wastewater (Kim et al., 2019). L. Bouarab et al. (2004) found that M. pusillum can grow well under mixotrophic conditions. It may provide opportunities for the industrial cultivation of microalgae and achieve high culture densities. Some studies have confirmed the suitability of Micractinium sp. for biofuel production (Abou-Shanab et al., 2014; Onay et al., 2014).

During the study on algal diversity of pyroclastic deposits of the Kamchatka Peninsula, Chlorella-like strains of green algae, previously identified as species of the genus Micractinium, were isolated. The aim of this study was species identification of Micractinium representatives using an integrative approach.

## Materials and methods

**Fig. 1.**
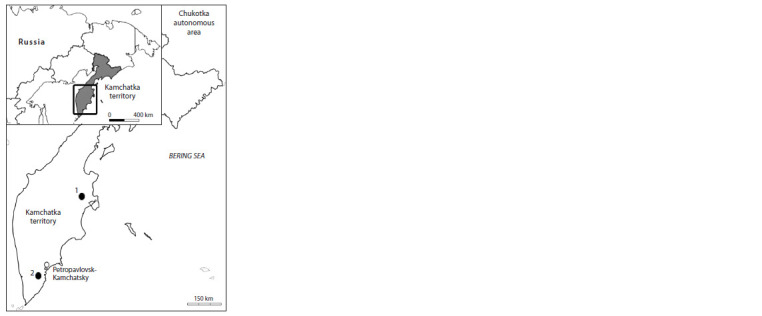
Map of the study area and sampling sites: 1 – Shiveluch volcano, 2 – Gorely volcano.

Sampling, isolation and cultivation of algal strains. The materials for this study were Chlorella-like clonal cultures of green algae VCA-72 and VCA-93. Strain VCA-72 was isolated from a sample of pyroclastic deposits collected in 2018 along the Baydarnaya river bed on the Shiveluch volcano (56°33.98′ N, 161°8.41′ E). Strain VCA-93 was detected from a sample collected in 2020 at the outlet of thermal vapors along the edge of the caldera on the southern slope of the Gorely volcano, where the temperature of the deposits was ~32 °C, (52°33.306′ N, 158°01.742′ E) (Fig. 1). The biotope on the Gorely volcano was characterized by a lack of vegetation. Melting of nearby snowfields and evaporation of moisture were observed during sampling. Sampling was carried out using classical microbiological methods (Gollerbah, Shtina, 1969).

A soil sample weighing not more than 1 g was inoculated on Petri dishes with sterile liquid modified Waris-H medium (McFadden, Melkonian, 1986; Andersen, 2005) (Supplementary Material 1)1 and liquid modified Bold Basal Medium with triple nitrogen and vitamins (Starr, Zeikus, 1993; Schlӧsser, 1997; Andersen, 2005) (Supplementary Material 1) to obtain enrichment cultures. Enrichment cultures were periodically checked for algal growth using an Olympus CK30 inverted microscope (Olympus, Japan) with a maximum magnification of ×400.


Supplementary Materials are available in the online version of the paper: https://vavilov.elpub.ru/jour/manager/files/Suppl_Sushchen_Engl_28_7.pdf


Pure cultures were isolated using the micropipette method (Andersen, 2005) and grown in modified Waris-H liquid medium. Algal cultures were maintained at 117–120 lux illumination, 24.9 °C, 16 % humidity, and 16:8 h light:dark cycle.

Light microscopy, morphological characterization. The morphology of the strains was examined with Olympus BX 53 (Olympus, Japan), equipped with Nomarski DIC optics. Microphotographs were taken with an Olympus DP 27 camera (Olympus, Japan) at ×1000 magnification. The parameters of 50 vegetative cells were analyzed to identify the boundaries of variation in morphological characteristics for each strain.

Molecular genetic analysis. Taxonomic identification of strains was performed by molecular genetic methods, including obtaining nucleotide sequences of the small subunit and internal transcribed spacer rRNA (18S+ITS rRNA; according to the protocol outlined by V.Yu. Nikulin et al. (2023)), construction of phylogenetic trees and secondary structures of the ITS1 and ITS2 rRNA regions.

For DNA isolation, cell biomass was sampled during the exponential growth phase and concentrated by centrifugation. Total genomic DNA was isolated according to the method of C.S. Echt et al. (1992) with some modifications (Abdullin et al., 2021). Amplification was performed by polymerase chain reaction (PCR) in a T100 Thermal Cycler amplifier (Bio-Rad Laboratories, Inc., USA) with Encyclo Plus kit (Evrogen, Russia), primers 82F (5′-GAAACTGCGAATGGCTC-3′) (López-García et al., 2003) and ITS4R (5′-CCTCCGCT TATTGATATGC-3′) (White et al., 1990). PCR parameters were as follows: initial denaturation at 96 °C for 3 min, followed by 30 cycles including denaturation at 96 °C for 1 min, annealing at 55 °C for 2 min, elongation at 68 °C for 3 min. This was followed by a final elongation at 68 °C for 7 min (Mikhailyuk et al., 2018).

Sequencing was performed using the equipment of the Instrumental Centre of Biotechnology and Gene Engineering of FSCEATB FEB RAS, ABI 3500 genetic analyzer (Applied Biosystems, USA). PCR products were sequenced in both directions with BigDye Terminator sequencing kit v. 3.1 (Applied Biosystems, USA) and the same primers as used for PCR. Additionally, primers SSU528F-800 (5′-CGGT AATTCCAGCTCC-3′) (Hoef-Emden, Melkonian, 2003), 920F (5′-GAAACTTAAAKGAATTG-3′) (Marin et al, 1998), n1400R (5′-GGTAGGAGCGACGGGCGGTGTGTAC-3′) (Marin et al., 2003), and Bd18SF1 (5′-TTTGTACACACCG CCCGTCGC-3′) (Goka et al., 2009) were used. Sequences were assembled with the Staden v.1.4 software package (Bonfield et al., 1995) and compared with other strains available at the National Center for Biotechnology Information (NCBI, USA) using a BLAST search (https://blast.ncbi.nlm.nih. gov/Blast.cgi). The selection of representative sequences for phylogenetic analysis was based on a dataset of green algae of the genus Micractinium (Krivina et al., 2023), which included 48 18S+ITS rRNA sequences; 2,418 aligned positions. The sequence of the taxon Chlorella vulgaris Beijerinck, representing a phylogenetically distant lineage, was added to the dataset as an outgroup. Sequences were aligned in the SeaView program (Galtier et al., 1996) with manual alignment correction. The best-fit model of nucleotide substitutions for our dataset was determined based on the Akaike Information Criterion (AIC) (Akaike, 1974) in the jModelTest 2.1.1 program (Darriba et al., 2012).

Phylogenetic trees were constructed using the maximum likelihood (ML) method and the Bayesian approach (BI). ML analysis was performed using the RAxML web server v. 7.7.1 (http://embnet.vital-it.ch/raxml-bb/) (Kozlov et al., 2019); BI was performed using the MrBayes 3.1.2 program (Huelsenbeck, Ronquist, 2001). In BI analysis, 5 million generations of Markov chains were created, sampling every 100 generations, i. e., 50,000 samples. The first 25 % of samples (before -lnL values reached a plateau) were excluded from the analysis as “burn-in”. Markov chain Monte Carlo convergence (MCMC) to a stationary distribution was assessed visually using the Tracer 1.7.1 program (Rambaut et al., 2018) by plotting posterior probabilities. All ESS values were greater than 200. The stability of ML-derived phylogenetic tree nodes was calculated using the RAxML server using the bootstrap method (Bootstrap Percentage, BP) (Stamatakis et al., 2008) and by determining the posterior probabilities (PP) in the BI. BP values less than 50 % and PP values less than 0.95 were not considered. Phylogenetic trees were visualized using the FigTree v. 1.4.4 program (Rambaut, 2018).

To confirm strain identification, secondary structures of the ITS1 and ITS2 rRNA regions were compared between phylogenetically related sequences. Secondary structures were constructed based on models developed for representatives of the genus Micractinium (Chae et al., 2019; Krivina et al., 2023) using the UNAFold Web Server (http://www.unafold. org/mfold/applications/rna-folding-form.php) (Zuker, 2003) and visualized in the VARNA program (Darty et al., 2009). Next, compensatory and hemicompensatory base substitutions (CBC, hCBC) (Caisová et al., 2013) and other molecular features that distinguish strains were searched for.

## Results and discussion

Molecular genetic analysis

The sequences of the region comprising 18S–ITS1–5.8S–ITS2 rRNA of strains VCA-93 and VCA-72 were deposited in the GenBank database under accession numbers PP501334 and PP501335, respectively. BLAST searches revealed a high percentage of similarity to the sequences of Micractinium sp. (M. thermotolerans) (Krivina et al., 2023) ACSSI 332 MT784118 (99.91 %) and M. inermum NLP-F014 KF597304 (99.29 %), respectively.

**Fig. 2.**
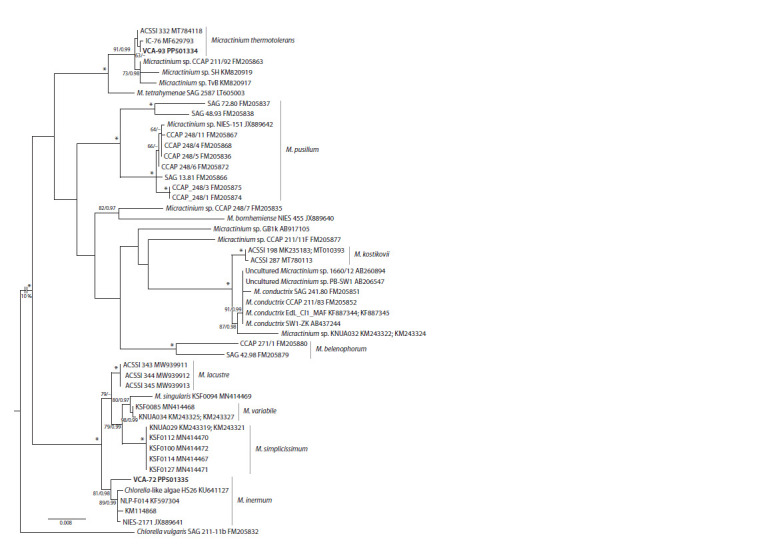
ML tree illustrating the phylogenetic position of strains VCA-93 and VCA-72 (in bold) among members of the genus Micractinium based on 18S+ITS rRNA sequence comparison (2,418 aligned positions; GTR+I+G model). Node supports in ML/BI analyses (BP ≥ 50 % and PP ≥ 0.95) are indicated. Nodes with maximum support (100/1.00) are indicated by asterisks. The branch belonging to the outgroup is shortened (only 10 % of the length is shown). Scale bar is the number of nucleotide substitutions per position.

On the phylogenetic tree, strain VCA-93 was clustered in the topologically established species clade of M. thermotolerans with strains ACSSI 332 (holotype) and IC-76 (Fig. 2). All three strains were found in the Russian Federation (Kamchatka Peninsula, Chukotski Peninsula (Krivina et al., 2023), and West Siberian Plain (Piligaev et al., 2018), respectively). Related to them is a clade with moderate statistical support (73/0.98; BP/PP) including strains of Micractinium sp. from Africa – TvB (isolated from Tiberias hot springs), SH (from a sinkhole near Ein Gedi), CCAP 211/92 (soil sample from Seychelles).

As noted earlier, in contrast to “African” strains, all representatives of M. thermotolerans are characterized by the absence of an intron in the second quarter of the 18S gene (Krivina et al., 2023). This is also true for strain VCA-93. The sister species was M. tetrahymenae SAG 2587.

Strain VCA-72 occupied a basal position in the moderately supported clade of M. inermum (81/0.98) (Fig. 2). In sister position was a clade (89/0.99) composed of closely related strains found in North America, Europe, and Asia: HS26 (Sonora Desert in Arizona, USA) (Ganuza et al., 2016), NLP-F014 (Nakdong River, South Korea) (Park et al., 2015), KM114868 (Weston Park Pond, UK) (Smith et al., 2015), and NIES-2171 (Sendai Botanical Garden, Japan) (Hoshina, Fujiwara, 2013). Thus, there was no geographic structuring in the M. inermum clade. The species clades of M. lacustre, M. variabile, M. simplicissimum and one specimen of M. singularis were the closest to the M. inermum clade.

ITS1 and ITS2 rRNA secondary structures

The secondary structures of the ITS1 and ITS2 regions of the studied strains corresponded to the generally accepted models developed for eukaryotic organisms, in particular, green algae (Coleman, 2000, 2015). The models of ITS1 and ITS2 helical domains of strain VCA-93 and M. thermotolerans strains ACSSI 332 and IC-76 (Supplementary Material 2) were characterized by the absence of substitutions between them.

**Fig. 3.**
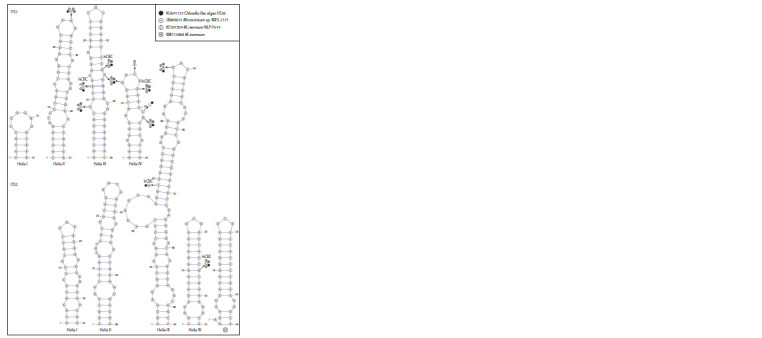
Secondary structure models of the ITS1 and ITS2 helical domains of strain VCA-72. Nucleotide sequence differences with M. inermum strains HS26, NLP-F014, KM114868, and NIES-2171 are indicated by endnotes according to the legend. Helix IV ITS2 of strain KM114868 is shown separately (bottom right) due to topological differences.

The presented models of the secondary structure of the helical domains of strain VCA-72 were generally similar to those for M. inermum (Fig. 3).

Helix I in ITS1 and helix I, II in ITS2 were monomorphic, but intraspecific variability was observed in all other ITS helices of the compared M. inermum strains. Despite this, the conservatism of the structure is due to the predominant localization of substitutions in apical or lateral loops and the presence of hCBCs that preserve base pairing. In terms of the number of nucleotide differences, the ITS1 regions were expectedly less conservative compared to ITS2 (ten versus four differences). The majority of nucleotide substitutions in both spacers (ten substitutions) distinguished our strain from the other four, but there were four substitutions and deletions characterizing specific strains (Fig. 3). Five hCBC substitutions were detected in the helixes (three hCBCs in ITS1: U→C in pos. 12 and 34 of helix III, A→G in pos. 17 of helix IV; two hCBCs in ITS2: C→U in pos. 29 of helix III, U→C in pos. 26 of helix IV), whereas no CBC substitutions were detected. The topology of the basal part of helix IV ITS2 strain KM114868 differed from the others (Fig. 3).

According to the CBC concept (Wolf et al., 2013), the absence of CBC in ITS2 indicates that the compared strains belong to the same species. Thus, based on the results of phylogenetic analysis and modelling of secondary structures, we reliably identified the strains under study: VCA-93 belongs to the species M. thermotolerans Krivina, Sinetova, Savchenko, Degtyarev, Tebina et Temraleeva, and VCA-72 belongs to M. inermum. The studied genotype of the latter, due to the presence of unique nucleotide substitutions, allowed us to add new data to the pool of molecular diversity of the ITS rRNA region of M. inermum species.

Morphology, reproduction and ecology

**Fig. 4.**
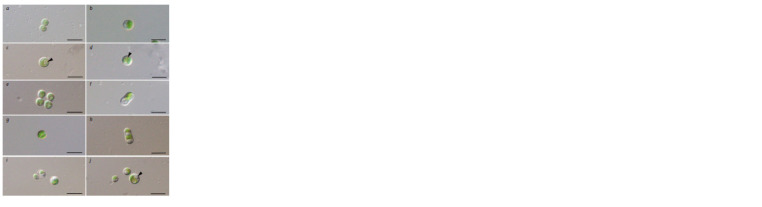
Microphotographs of the strains M. thermotolerans VCA-93 (a–f ) and M. inermum VCA-72 (g–j). a, b – vegetative cells; c – presporangial cell (arrow indicates doubling of the pyrenoid); d – presporangial cell (arrow indicates doubling of the protoplast); e – autosporangia; f – ellipsoid cell with a shallow, wide constriction in the center with vacuoles; g – vegetative cell; h – sporangium in an aging culture; i – release of autospores; j – cell in an aging culture with a lipid droplet and vacuole (lipid droplet is indicated by an arrow). Scale bar: 10 μm.

Micractinium thermotolerans Krivina, Sinetova, Savchenko, Degtyarev, Tebina et Temraleeva (Fig. 4а–f ). The cells are spherical, 3.2–6.5 μm in diameter, without bristles (Fig. 4a, b). Young cells are triangular, ellipsoidal (3.0–5.6 × 3.3–5.9 μm) or irregular. The chloroplast is parietal, cup-shaped with a spherical pyrenoid, covered by starch grains. Reproduction by 2–4 autospores (Fig. 4c–e). The sporangium size was 4.4–6.7 μm in diameter. Autospores were uniform in size (up to 2 microns in diameter), triangular or irregular in shape and showed release by rupture of the sporangium cell wall. Cell walls remain visible in culture after release of autospores.

The cell wall is thin, with uniform thickening in older cultures. The cells are pear-shaped, oval and ellipsoidal with a shallow, wide constriction in the center, reaching a length of 7.3–10.5 μm in 6-month cultures (Fig. 4f ).

The detection of cells significantly larger than mature vegetative cells is consistent with the observations of E. Krivina et al. (2023). They showed that incubation at elevated, but non-lethal temperatures caused the appearance of a population of single or abnormally dividing giant cells with a diameter of 10.8–19.3 μm in the culture. In our case, the M. thermotolerans VCA-93 strain was cultured at 24.9 °C, and the appearance of abnormal cells with lipid droplets was probably a result of culture depletion. It was associated with its aging. It is similar to the results obtained for M. thermotolerans ACSSI 332 under nitrogen starvation conditions.

E. Krivina et al. (2023) obtained preliminary data on the fatty acid composition of M. thermotolerans. Thus, during the description of the species, the composition of methyl esters of fatty acids of strain ACSSI 332 was revealed (hexadecanoic, 7,10,13-hexadecatrienic, 9,12,15-octadecatrienic, pentadecanoic acids, etc.). It differed significantly from the fatty acid composition of other species of the genus Micractinium with greater complexity and diversity (Krivina et al., 2023). The authors noted the biotechnological potential of this species.

Two of the three known strains of M. thermotolerans were isolated from extreme habitats: VCA-93 from tephra collected at the outlet of thermal vapors along the edge of the caldera of the Gorely volcano (Kamchatka Peninsula) and ACSSI 332 from a hot spring located on the Chukotka Peninsula (Krivina et al., 2023). At the same time, strain IC-76 was isolated from river sand from the coast of the Ob river (Novosibirsk region) (Piligaev et al., 2018), which may indicate the ecological plasticity of the species.

Micractinium inermum Hoshina et Fujiwara (Fig. 4g–j). The solitary cells are spherical (4.3–5.0 μm) (Fig. 4g), drop-shaped or ellipsoidal (2.2–4.7 × 3.0–5.0 μm), without bristles. The chloroplast is single, cup-shaped, with a pronounced pyrenoid. Asexual reproduction by two autospores (Fig. 4i). Cells in old cultures are spherical (5.7–7.9 μm) or ellipsoidal with a shallow, wide constriction in the center (Fig. 4h), 8.4–10.7 μm long and are characterized by the presence of lipid droplets proportional to their size (Fig. 4i, j).

In the cytoplasm of aging and resting cells, there is an accumulation of single small or large lipid droplets (Andreeva, 1998). It can be colorless or yellow, orange, red. Probably, the color of lipid droplets is associated with carotenoids and their derivatives, which are accumulated in lipid globules. They can be detected by light microscopy in the form of spherical colored bodies in the resting stages of many green algae species (Weiss, 1983). For example, it has been shown that the reddish color of lipid droplets of Haematococcus pluvialis Flotow is due to the presence of the fat-soluble carotenoid astaxanthin (Ota et al., 2018). According to our observations, aging cells of M. inermum VCA-72 are characterized by the presence of colorless lipid droplets (Fig 4h–j).

The detection of lipid droplets in cells is also characteristic of culture depletion, including nitrogen starvation. For example, J. Zhan et al. (2016) found significant changes in the lipid content of Chlorella sp. under nitrogen depletion of the culture. Nutrient deficiency, as well as high light intensity and high salt concentration, is an environmental stressor and causes the accumulation of lipids or carbohydrates (Ho et al., 2012; Fernandes et al., 2013; Roleda et al., 2013; Park et al., 2015).

It is known that the Micractinium species studied by us are characterized by high growth rates and the ability to synthesize and accumulate lipids (Park et al., 2015; Shi et al., 2019; Krivina et al., 2023). This aspect indicates their great potential for the production of biodiesel (Wijffels, Barbosa, 2010). There have been a number of works aimed at identifying the optimal culture conditions for M. inermum, which allow increasing its biological productivity and reducing the cost of maintaining cultures. For example, it has been shown that the maximum lipid productivity of the JL1 strain is achieved by adding glucose to the heterotrophic culture (Shi et al., 2019). The fatty acid profile of algae is of great importance in biodiesel production as it determines the key properties of the fuel (Knothe, 2009). For example, the percentage of oleic acid serves as an indicator of fuel quality (Knothe, 2009). A. Banskota et al. (2024) identified oleic, linoleic and palmitic acids in the biomass of M. inermum.

S. Park et al. (2015) suggested the use of wastewater mixture for M. inermum NLP-F014 cultivation. The lipid accumulation was up to 40 % under culture depletion conditions. This method can significantly reduce the cost of water and nutrient requirements, and hence cost of cultivation. T. Sydney et al. (2018) treated M. inermum culture with ultraviolet B (UVB) to reduce the energy required for cell wall disruption and lipid extraction. This resulted in an increased yield of fatty acid methyl esters. Thus, strains of this species are candidates for biofuel production

Most publications (Park et al., 2015; Smith et al., 2015; Dickinson et al., 2019; Shi et al., 2019; Banskota et al., 2024) indicate freshwater habitats for M. inermum. Probably, small sizes and rapid reproductive rates allow representatives of this species not only to be planktonic in water bodies, but also to survive in terrestrial habitats, including in such extreme biotopes as the volcanic deposits of Kamchatka (pyroclastic deposits along the Baydarnaya river bed on the Shiveluch volcano).

## Conclusion

As a result of the study of algal diversity in the pyroclastic deposits of the Shiveluch and Gorely volcanoes (Kamchatka Peninsula), using an integrative approach, representatives of the genus Micractinium were identified. The results obtained complement the information on the secondary structure of the ITS1 and ITS2 rRNA regions, morphology (morphology of cells in aging cultures of M. inermum and M. thermotolerans), life cycle (the life cycle of M. inermum is considered in more detail), ecology (the species are among the primary colonizers of lifeless substrate on the Kamchatka Peninsula; vital activity of M. thermotolerans at deposit temperature ~32 °C) and biogeography (M. inermum is reported for the first time in Russia, and M. thermotolerans is the first finding for the Kamchatka Peninsula) of the discovered species.

## Conflict of interest

The authors declare no conflict of interest.
